# Pamphlets Received

**Published:** 1854-08

**Authors:** 


*Transactions of the Medical Association of the State of Alabama.* 1854.

The Medical Association of Alabama seems to be in a healthy and active condition. Among the many valuable papers in this volume, we notice one on the " Unity of Disease," by H. Backus, M. D. It is a continuation of an essay read at a previous meeting, and which we had occasion to notice favorably nearly a year since. It is not within our power at present to say more of this essay. It is replete with thought, and evinces a capacious power of generalization. Whether or no congestion be the essential unit of all disease, we cannot affirm; but we can readily recognize in this paper a wide collection of relevant facts, and a strong argument, which go far toward proof of Dr. Backus's doctrine.

*Essay on the Mechanism and Management of Parturition, in the Shoulder Presentation.* By Wm. M. Boling, M. D., of Montgomery, Alabama.

This is a very well written treatise of some ninety pages. Dr. Boling handles his subject with much skill and learning. He seems to regard spontaneous evolution as a more frequent occurrence than it is generally considered. We recollect an instance when the practitioner found the arm prolapsed, and everything crowded forcibly down by very vigorous pains. He gave a five grain dose of opium, and left the house to send for a consulting accoucheur. On his return, after a short absence, he found the breech presenting, and the labor soon terminated favorably. Such accidents, however, should not be relied on.

*Steps to the Medical Platform.* An Address, by J. Adams Allen, M. D., Prof. of Physiology, &c., in the Michigan University.

The object of this address seems to be to make some necessary repairs in that very doubtful platform which its author constructed last winter. The sins of that essay should not, however, be visited upon this address, which possesses a far more moderate tone, and is not wanting in orthodoxy. We learn that the connection of Prof. Allen with the Michigan University has been recently discontinued.

*Summary of the Transactions of the College of Physicians of Philadelphia—from Nov.* 2, 1853, *to Jan.* 4, 1854.

As usual, these Transactions are very interesting. The names of Jackson, of Northumberland, Stillé, Parish, Ruschenberger, and Hewson, are a few of the working members of this working society.

*On the subject of Priority in the Medication of the Larynx and Trachea.* By Horace Green, M. D.

This vexed question is here again brought before the public, in the form of a defense of the author from the attacks of an American physician in France. Dr. Green vindicates himself with much ability; but the matter has taken so purely personal a shape, that we must avoid any discussion of its merits.

